# Adrenal Incidentalomas in Cancer Patients Are Not Always “Innocent”: A Case Report and Review of the Literature

**DOI:** 10.1155/2013/461409

**Published:** 2013-04-10

**Authors:** Panagiota Economopoulou, Giannis Mountzios, Ioannis Kotsantis, Marios Bakogeorgos, Vassilios Ramfidis, Ioannis Kapiris, Efstratios Patsouris, Nikolaos Kentepozidis

**Affiliations:** ^1^251 Airforce General Hospital, Medical Oncology Department, 3RD P. Kanellopoulou Street, Katehaki, 11725 Athens, Greece; ^2^251 Airforce General Hospital, Medical Oncology Department, 2nd Department of Surgery, 3RD P. Kanellopoulou Street, Katehaki, 11725 Athens, Greece; ^3^University of Athens, Department of Pathology, 75th Mikras Asias Street, Goudi, 11527 Athens, Greece

## Abstract

Herein, we report an unusual case of a 78-year-old woman with synchronous presentation of sigmoid cancer and a nonfunctioning primary adrenal cortex carcinoma, who developed superior vena cava syndrome due to metastatic lymphadenopathy from the latter malignancy. Our case suggests that adrenal incidentalomas during initial staging evaluation after cancer diagnosis are not always “innocent” and should not be “*a priori*” considered incidental findings attributed to hyperplasia, adenoma or even a non life-threatening metastasis from the primary tumor. It also emphasizes the importance of a continuous assessment of patients with synchronous primary malignancies, in order to timely evaluate changes in clinical or biological behavior and administrate the appropriate treatment.

## 1. Introduction

Primary carcinoma of the adrenal cortex (ACC) is a rare and highly aggressive malignancy, accounting for an estimated 0.02% of all cancers [1]. Approximately 60% of ACCs are hormonally active, presented clinically as Cushing syndrome (in glucocorticoid excess), virilization (in androgen excess), or hypokalemia (in mineralocorticoid excess). In contrast, hormonally inactive ACCs usually present with abdominal discomfort (nausea, vomiting, and abdominal fullness) or back pain caused by a mass effect of a large tumor [1]. Data from case series indicate that the majority of patients with ACC present with regional or distant spread; common sites of metastasis involve the liver, lungs, distant lymph nodes, and bone [2].

ACC accounts for <5% of all adrenal lesions detected incidentally on radiographic imaging, referred to as “adrenal incidentalomas” [3]. In patients with malignancy, the approach to incidentaloma is more critical because several malignant neoplasms have the tendency to metastasize to adrenal glands, the most notorious being non-small cell lung cancer. In this setting, distinction between metastasis, benign adrenal lesions, or primary ACC is difficult with CT or MRI imaging [4, 5]. Herein, we present an unusual case of a patient with colon cancer presented with an adrenal incidentaloma on preoperative imaging that was proved to be a nonfunctional ACC.

## 2. Case Presentation

A 78-year-old female was referred to our institution after synchronous resection of cancer of the sigmoid colon and a left adrenal lesion. The patient initially sought evaluation at a local clinic with a 2-month history of change in bowel movements, blood in the stool, and abdominal pain. She had no remarkable medical history and was under no medications at the time. A colonoscopy performed at the same institution revealed a hemorrhagic mass measuring 5 cm at the sigmoid region; subsequent biopsy revealed a moderately differentiated adenocarcinoma. Preoperative staging including computed tomographic (CT) scan of the abdomen, revealed a 13.5 × 8.5 × 7.6 cm adrenal mass with heterogeneous enhancement. A whole-body positron emission tomography (PET)/CT scan was performed, showing significant ^18^F-fluorodeoxyglucose (^18^F-FDG) uptake in the sigmoid colon and the left adrenal gland. No additional sites with increased uptake were identified.

**Figure 1:**
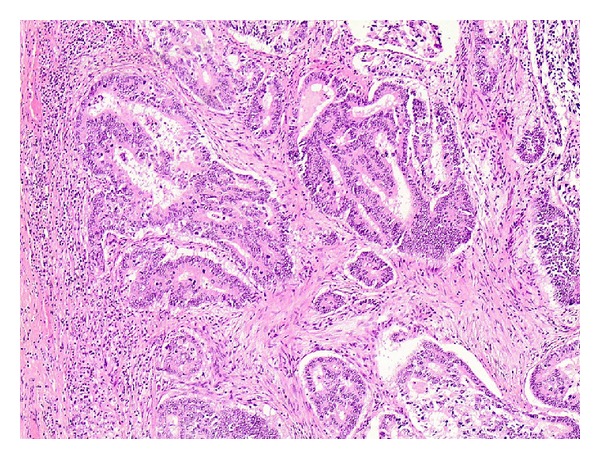
Adenocarcinoma of the colon, 100x magnification.

**Figure 2:**
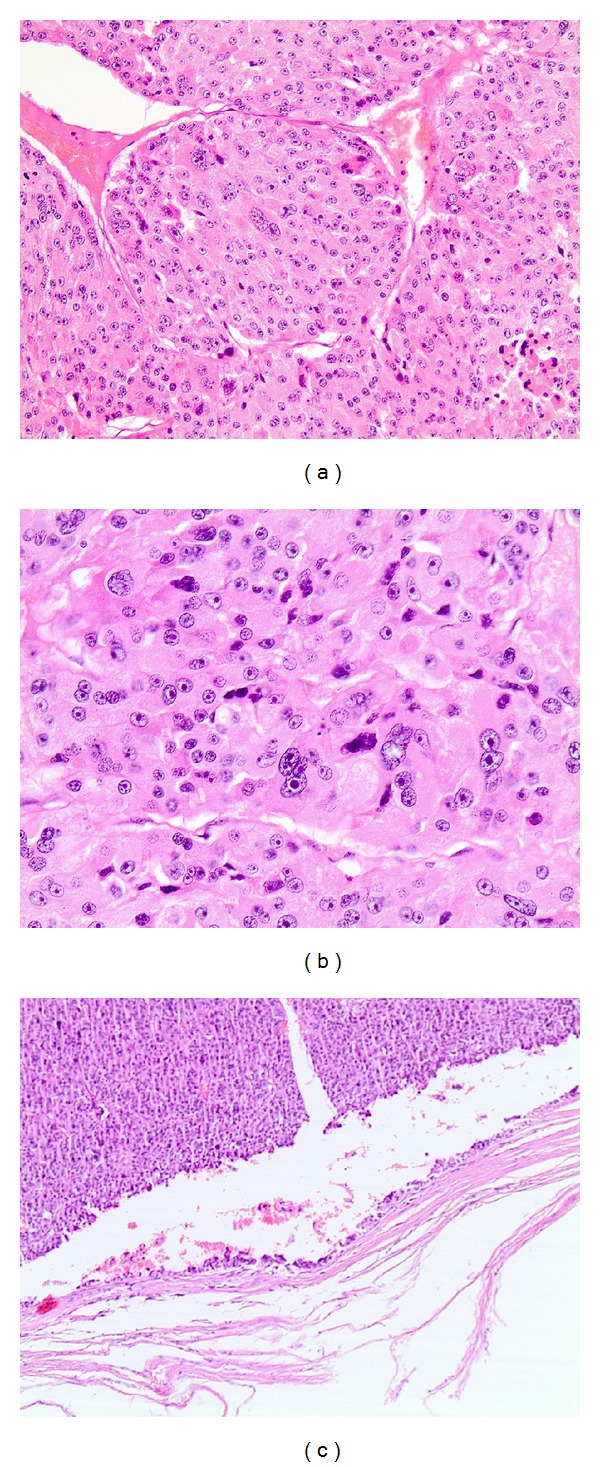
Adrenal carcinoma. (a) 200x magnification. (b) Pathologic view showing atypical mitoses, 400x magnification. (c) Pathologic view demonstrating capsular invasion, 100x magnification.

**Figure 3:**
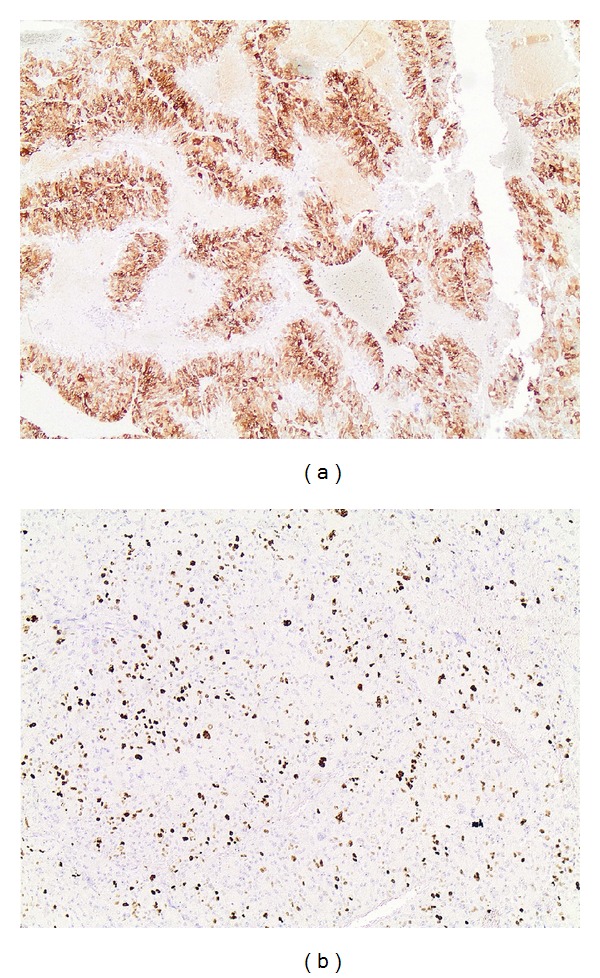
Immunohistochemical staining of adrenal carcinoma. (a) Synaptophysin positivity. (b) Positivity for mitotic index Ki-67%.

Since ^18^F-FDG-PET scan suggested malignant potential of the adrenal lesion, a synchronous resection of both sigmoid and adrenal lesion was opted. By virtue of the unknown origin of the adrenal mass, a thorough endocrine analysis, as proposed by European Network for the Study of Adrenal Tumors (ENSAT) [6], was made before resection. Blood pressure and potassium levels were within normal limits. Serum glucose, plasma ACTH, and dexamethasone suppression test, measured to assess glucocorticoid excess, were also within normal range. Determination of metanephrines in the urine excluded the presence of a pheochromocytoma. Due to patient's hirsutism, serum dehydroepiandrosterone sulphate (DHEA-S) was also measured, which was within the normal limits. Sigmoidectomy and locoregional lymph node dissection revealed a stage IIIB (T3N1M0) adenocarcinoma of the colon (Figure 1), while resection of the adrenal mass disclosed a stage III (T3N0M0) adrenal carcinoma with Weiss score of 4 [7], a mitotic rate of 10 per 50 high power fields (HPF), atypical mitoses, and capsular invasion (Figures 2(a), 2(b) and 2(c)). Immunohistochemical analysis of the biopsy specimens revealed a positive staining for synaptofphysin (Figure 3(a)) and Ki-67 mitotic index (15%—Figure 3(b)). Tumor markers, including CEA and CA-19-9, were within normal limits.

At that time, the patient was referred to our institution for further evaluation and treatment. Given the absolute benefit offered by adjuvant chemotherapy in patients with stage III colon cancer [8], whereas data regarding the use of mitotane in the adjuvant setting of ACC is still controversial [2], she received adjuvant chemotherapy with the FOLFOX regimen (Oxaliplatin 85 mg/m^2^, Leukovorin 200 mg/m^2^, 5-FU 400 mg/m^2^ bolus, 5-FU 600 mg/m^2^ continuous infusion for 46 hours). After 2 months (8 cycles) of treatment, the patient was re-evaluated with CT imaging of the chest and abdomen, which indicated a new 17 mm nodular mass in the azygoesophageal recess and enlarged right lower paratracheal lymph nodes. We decided to perform both a biopsy of the mass in the azygoesophageal recess and of the lower paratracheal lymph node mass, in order to identify the site of origin. Biopsy of both lesions suggested ACC as the primary origin of the lymph node involvement. Consequently, first-line treatment for advanced ACC was started consisting of cisplatin 40 mg/m^2^, adriamycin 40 mg/m^2^, etoposide 100 mg/m^2^, and oral mitotane initially at a dose of 2 g/day with continuous dose monitoring; hydrocortisone replacement therapy 50 mg/day was added. During treatment with mitotane, full blood count, liver function tests, ACTH, thyroid hormones, cholesterol, and renin were regularly measured. The drug was well tolerated and gradually increased at a final dose of 12 g/day. Chemotherapy was discontinued after 6 cycles, due to platinum-related sensorineural hearing loss and retinal detachment, for which she was successfully treated with vitrectomy, resulting in gradual vision gain. Of note, re-evaluation after the completion of 3 cycles of chemotherapy, had shown stabilization of the disease (modified RECIST criteria).

Three months after cessation of chemotherapy the patient presented at the emergency room with worsening dyspnea, swelling of the face and neck, and dilatation of veins on her upper chest. Electrocardiogram indicated paroxysmal atrial fibrillation (AF). The patient also underwent a CT scan of the chest, which revealed enlargement of the paratracheal nodes with signs of obstruction of superior vena cava. The patient underwent chemical cardioversion of AF, while administration of mitotane was reduced progressively. She was promptly started on conventional radiotherapy, which resulted in shrinkage of the size of lymph nodes and subsequent improvement of respiratory distress and facial edema. One month after completion of radiotherapy, second line chemotherapy with weekly paclitaxel was administered, while mitotane treatment was stopped. After completion of 6 weeks of therapy, further partial response in the mediastinum was noted. After 4 more cycles of paclitaxel treatment, the patient developed abdominal pain. CT imaging of the abdomen performed at that time showed deterioration with evidence of malignant peritoneal implantations. The case was discussed at the multidisciplinary oncology meeting; CT-guided needle aspiration biopsy of peritoneal lesions was proposed in order to define the origin of peritoneal carcinomatosis, but the patient refused to undertake the procedure. It was thus decided that treatment at that point should aim primarily at colon cancer, since peritoneal disease was more likely to represent a manifestation of colon rather that adrenal cancer. She was started on modified FOLFIRI-Bevacizumab regimen (Irinotecan 180 mg/m^2^, Leukovorin 200 mg/m^2^, 5-FU bolus 400 mg/m^2^, 5-FU infusional 600 mg/m^2^ and bevacizumab 5 mg/kg). Thus far, she has received 2 cycles of chemotherapy with clinical benefit, defined by elimination of abdominal pain.

## 3. Discussion

Herein, we describe an unusual case of ACC initially presented as an incidentaloma in a patient with colon cancer, which progressively displayed a metastatic behavior causing superior vena cava syndrome and upper airway obstruction.

Adrenal incidentalomas in cancer patients are not always “innocent”; ACC accounts for approximately 5% of adrenal incidentalomas [3]. However, in the majority of patients with malignancy, adrenal incidentalomas most commonly represent sites of metastases of the primary tumor or benign adrenal adenomas [9]. Furthermore, synchronous existence of colorectal and adrenal carcinoma is relatively uncommon. In two case series reported by a Chinese group that included 4 patients undergoing synchronous resection of colorectal cancer and adrenal lesion, a diagnosis of ACC was made in a single patient [10, 11]. Furthermore, numerous case series report on the pathological diagnosis and outcome of patients with malignancy undergoing adrenalectomy for incidentaloma; in a study evaluating the role of imaging and surgery in 42 patients with cancer, including colon cancer and adrenal lesions, none of the patients was found to have an ACC [12]. Similarly, in two series of a limited number of patients with malignancy subjected to laparoscopic adrenalectomy reported, no case of ACC was reported [13, 14]. Moinzadeh and Gill have reported a pathological diagnosis of ACC in 6/31 patients with malignancy, although the site of the coexisting tumor in patients with ACC is not clearly referred in the study [15]. However, in the majority of these studies, adrenal masses were discovered during followup for the primary malignancy, which does not allow direct comparison with the case of our patient.

Superior vena cava syndrome (SVC) is considered a medical emergency; in few cases treatment can be delayed, until a histologic diagnosis is obtained. In the case of our patient, histopathological diagnosis was made before superior vena cava was compressed by the increasing size of the mediastinal lymph nodes. Although regional lymph node metastasis is encountered in 24% of patients with ACC, metastasis to mediastinal lymph nodes is rare [2]. SVC syndrome directly related to metastatic ACC has been reported only once in the literature [16]. The patient was treated with radiation, since ACC is not considered a chemosensitive tumor; chemotherapy with cisplatin, adriamycin, and etoposide had already been administered in our patient without evidence of objective response.

The choice of treatment in a patient with two synchronous cancers is a difficult task. We selected to treat the patient with adjuvant chemotherapy for her colon cancer, considering the high risk of relapse in stage III colon cancer [8]. On the other hand, there is an ongoing debate as to whether patients benefit from adjuvant mitotane in ACC. In patients with localized ACC and R0 resection, adjuvant therapy is suggested if mitotic index Ki67 is >10% and tumor size is >8 cm [6]. In the case of our patient, Ki67% was 20% and tumor size 13.5 cm in major dimension, but the risk for developing distant metastases was statistically higher for colon cancer than for ACC and the hypothetic combination of the two regimens was expected to be highly toxic, particularly in terms of myelosuppression. It is unclear at the moment whether administration of adjuvant mitotane would confer any substantial clinical benefit. Nevertheless, once the diagnosis of metastatic ACC was established, adjuvant chemotherapy for colon cancer was discontinued, and first-line chemotherapy for the advanced ACC was initiated. Given the rarity of the tumor and the paucity of clinical trials, second line chemotherapy for ACC (weekly paclitaxel) was chosen based on published case series [17].

**Figure 4:**
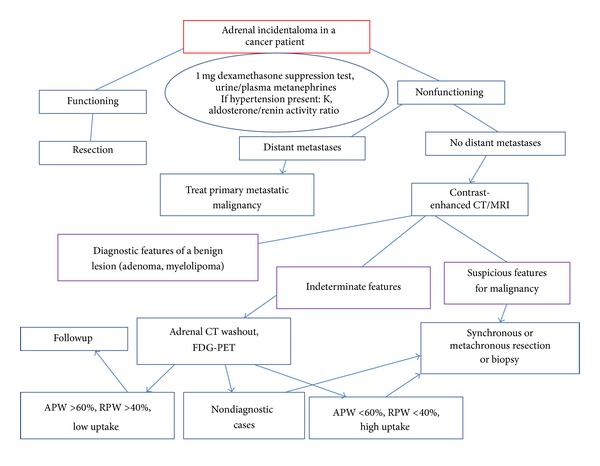
Proposed algorithm for the management of cancer patients with adrenal incidentaloma. An adrenal CT washout is a triphasic adrenal CT used for determining whether an adrenal lesion is an adenoma. It is based on the fact that adenomas (regardless of lipid content) show pathognomonic rapid enhancement and corresponding rapid washout following contrast medium administration. CT: Computed Tomography, MRI: Magnetic Resonance Imaging, RPW: Relative Percentage Washout, APW: Absolute Percentage Washout, PET: Positron Emission Tomography.

On the other hand, it is worth mentioning that the adrenal lesion described in our report was, based on its size and imaging features, highly suspicious for malignancy; therefore, surgical resection was ab initio indicated. On the contrary, adrenal incidentalomas with indeterminate features on CT imaging can pose a therapeutic dilemma in the management of cancer patients treated with curative intent. A diagnostic algorithm for the evaluation of adrenal incidentaloma in patients with known malignancy is proposed in Figure 4.

In conclusion, we report an unusual case of a patient with synchronous presentation of nonfunctioning ACC and colon cancer, who developed SVC syndrome due to metastases from ACC. Our case suggests that adrenal incidentalomas are not always “innocent” and should not be “a priori” considered incidental findings related to hyperplasia or adenoma. It also emphasizes the importance of a continuous assessment of patients with synchronous primary malignancies, in order to timely evaluate changes in clinical or biological behavior and administrate the appropriate treatment.
